# Clinical and Translational Imaging and Sensing of Diabetic Microangiopathy: A Narrative Review

**DOI:** 10.3390/jcdd10090383

**Published:** 2023-09-06

**Authors:** Nikolina-Alexia Fasoula, Yi Xie, Nikoletta Katsouli, Mario Reidl, Michael A. Kallmayer, Hans-Henning Eckstein, Vasilis Ntziachristos, Leontios Hadjileontiadis, Dimitrios V. Avgerinos, Alexandros Briasoulis, Gerasimos Siasos, Kaveh Hosseini, Ilias Doulamis, Polydoros N. Kampaktsis, Angelos Karlas

**Affiliations:** 1 Institute of Biological and Medical Imaging, Helmholtz Zentrum München, 85764 Neuherberg, Germany; nikolina.fasoula@tum.de (N.-A.F.); yixie555@gmail.com (Y.X.); nikoletta.katsouli@tum.de (N.K.); bioimaging.translatum@tum.de (V.N.); 2 Chair of Biological Imaging at the Central Institute for Translational Cancer Research (TranslaTUM), School of Medicine, Technical University of Munich, 81675 Munich, Germany; 3 Department for Vascular and Endovascular Surgery, Klinikum rechts der Isar, Technical University of Munich (TUM), 81675 Munich, Germany; michael.kallmayer@mri.tum.de (M.A.K.); hheckstein@web.de (H.-H.E.); 4 DZHK (German Centre for Cardiovascular Research), Partner Site Munich Heart Alliance, 80336 Munich, Germany; 5 Department of Biomedical Engineering, Healthcare Engineering Innovation Center (HEIC), Khalifa University, Abu Dhabi P.O. Box 127788, United Arab Emirates; leontios.hadjileontiadis@ku.ac.ae; 6 Department of Electrical and Computer Engineering, Aristotle University of Thessaloniki, 54124 Thessaloniki, Greece; 7 Onassis Cardiac Surgery Center, 17674 Athens, Greece; davgerinos@gmail.com; 8 Aleksandra Hospital, National and Kapodistrian University of Athens Medical School, 11527 Athens, Greece; alexbriasoulis@gmail.com; 9 Sotiria Hospital, National and Kapodistrian University of Athens Medical School, 11527 Athens, Greece; gsiasos@med.uoa.gr; 10 Cardiac Primary Prevention Research Center, Cardiovascular Disease Research Institute, Tehran University of Medical Sciences, Tehran 1411713138, Iran; kaveh_hosseini130@yahoo.com; 11 Department of Surgery, The Johns Hopkins Hospital, School of Medicine, Baltimore, MD 21287, USA; doulamis.i@gmail.com; 12 Department of Medicine, Columbia University Irving Medical Center, New York, NY 10032, USA; pkampaktsis@yahoo.com

**Keywords:** microvascular imaging, optoacoustics, ultrasound, MRI, complications of diabetes, optical imaging, molecular imaging

## Abstract

Microvascular changes in diabetes affect the function of several critical organs, such as the kidneys, heart, brain, eye, and skin, among others. The possibility of detecting such changes early enough in order to take appropriate actions renders the development of appropriate tools and techniques an imperative need. To this end, several sensing and imaging techniques have been developed or employed in the assessment of microangiopathy in patients with diabetes. Herein, we present such techniques; we provide insights into their principles of operation while discussing the characteristics that make them appropriate for such use. Finally, apart from already established techniques, we present novel ones with great translational potential, such as optoacoustic technologies, which are expected to enter clinical practice in the foreseeable future.

## 1. Introduction

Global rates of diabetes are significantly rising. In 2021, its prevalence was 537 million. This number is expected to increase to 643 million in 2030 [1]. Diabetes, a complex disorder where the body does not produce or properly use insulin, comes with metabolic dysregulations that cause vascular changes. In fact, persistently high blood glucose levels (the pathophysiological hallmark of diabetes), insulin resistance, and excess free fatty acids lead to micro- and macrovascular damage, affecting several ‘target’ organs, such as the peripheral nerves, brain, eyes, heart, kidneys, adipose tissues, skeletal muscles, and skin. 

Diabetic microangiopathy, which refers to pathologic changes of the microvessels (diameter less than 100–150 μm [2]), is also multifactorial and involves damage of the endothelium, inflammatory activation, microthromboses, and fibrosis. The main result of the previous processes is a decrease in tissue perfusion and oxygenation, functional degradation, and tissue damage. However, even if it seems that different vascular beds are affected in different organs and cause relevant complications (e.g., retinopathy and nephropathy are supposed to be mainly of microvascular origin, while heart disease is of macrovascular origin), there is a long-lasting debate about the pathophysiological ‘demarcation’ or interconnection between the micro- and macrovascular complications in diabetes [3]. For example, it is thought that microvascular changes, known as diabetic microangiopathy, promote atherosclerosis, the main cause of macrovascular disease. Thus, the thorough exploration of diabetic microangiopathy is of great importance not only for gaining insights into its pathophysiology or the pathophysiology of diabetes per se but also for detecting other complications early enough to take action and improve the prognosis. 

Diabetic microangiopathy cannot be directly detected via possibly relevant blood tests, which are often organ-specific (e.g., renal functional parameters, such as serum creatinine), but may only indicate functional degradation without identifying the underlying cause. Tissue biopsy is an ideal but invasive option that may provide an accurate diagnosis of organ-specific diabetic microangiopathy, but it is associated with complications such as hemorrhage and is not suitable for routine clinical practice. Non-invasive sensing and imaging techniques have been developed to detect and potentially quantify microangiopathy in diabetes. These techniques offer a safer and more practical alternative to tissue biopsy.

Different organs require specific techniques to assess organ-specific diabetic microangiopathy, depending on the features of the target organ and the properties of each technique. For example, ultrasonography (US), US-based elastography, laser Doppler flowmetry (LDF), magnetic resonance imaging (MRI), positron emission tomography (PET), optical coherence tomography (OCT), perfusion computed tomography (pCT), or nerve conduction studies (NCS) are some of the commonly used techniques. Herein, we provide an overview of the most common sensing and imaging techniques used to evaluate diabetic microangiopathy in different organs and tissues. We also include information on novel experimental/translational techniques, such as optoacoustics (raster-scan optoacoustic mesoscopy (RSOM) and multispectral optoacoustic tomography (MSOT)), which show great potential for clinical use in the near future within the field of metabolic disease [4,5,6,7,8]. A comparison of the main techniques discussed herein is provided in Table 1.

The presented study aims to provide a comprehensive overview of the subject by providing insights into the advantages and disadvantages of each technique, enabling clinicians and researchers to make decisions regarding the most appropriate diagnostic approach.

### Methodological Perspectives

Our work provides a narrative overview of the most characteristic clinical and translational imaging and sensing technologies for the assessment of diabetes-related microangiopathy in different tissues and organs. To provide an overview of the explored topic, we have employed two widely used research databases: PubMed and Google Scholar. For our search, we used the keywords ‘imaging diabetic microangiopathy’, ‘sensing and sensors for diabetic microangiopathy’, ‘imaging in diabetes’, ‘microvascular imaging in diabetes’, and ‘imaging microangiopathy in diabetes’. After retrieving the search results, we then selected the most relevant articles by means of a thorough hand search. Finally, we presented and discussed the selected studies. Taking into account that our review is narrative, the authors decided that following the PRISMA guidance was unnecessary.

## 2. Diabetic Nephropathy

Diabetic nephropathy (DN), or diabetic kidney disease (DKD), is the leading cause of mortality in patients with diabetes. DN is the diabetes-related progressive deterioration of renal function leading to chronic kidney disease (CKD) and, eventually, end-stage renal disease (ESRD) [9]. DN occurs in 20–40% of patients with diabetes [10]. In DN, processes such as inflammation, apoptosis, and endothelial dysfunction (ED) lead to microvascular degenerative changes such as thickening of the basement membrane, fibrosis, arteriosclerosis, atrophy of the kidney parenchyma, and loss of its functionality [11,12]. Although usual screening for DN is based on urine and blood tests, such as the levels of albuminuria and glomerular filtration rate (GFR) [13], renal imaging plays a key role in the diagnosis and monitoring of DN and its progression. 

US with color Doppler imaging (CDI) is the first-line technique used in the diagnosis of CKD. By generating, transmitting, and receiving the reflected US waves after their interaction with soft tissues and blood, US techniques manage to provide high-resolution (~1 mm) real-time visualizations of tissue structure and function via dynamic blood flow profile visualizations (CDI) without employing contrast agents or ionizing radiation. US may well be used not only to detect macroscopic renal alterations but also to differentiate between early- and late-stage changes. Thus, US studies show an increase in kidney volume and cortex thickness at the early stages of the disease [14], followed by decreased total size and cortex echogenicity as well as more echogenic renal parenchyma [15]. Yet, apart from only morphological or textural imaging, CDI can provide insights into functionality by measuring the renal resistance index (RI, defined as the ((peak systolic velocity—end-diastolic velocity)/peak systolic velocity) in the measured artery), which reflects the intrarenal vascular resistance, i.e., a feature that is particularly high in the main renal and interlobar arteries in patients with DN [16]. The intrarenal RI, in combination with the end-diastolic velocity (EDV), also shows significant diagnostic value for the early detection of DN [17]. Moreover, in [18], the potential value of three-dimensional US (3DUS) in the diagnosis of DN is demonstrated. 

**Figure 1:**
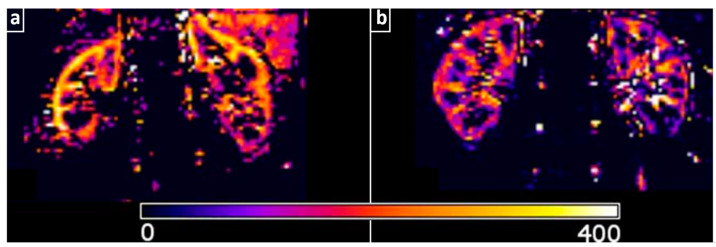
Imaging of nephropathy in diabetes. (**a**, **b**) Renal blood flow (RBF) maps of a representative healthy control (**a**) and a patient with T2DM (**b**) were measured with arterial spin labeling (ASL) MRI, showing lower perfusion in the patient’s RBF map. Adapted with permission from [23].

Apart from US, MRI techniques have also been used in imaging DN. In particular, functional MRI (fMRI) has been introduced as a tool to assess renal blood flow (RBF), parenchyma perfusion, and oxygenation, as well as degenerative processes such as inflammation and fibrosis, i.e., features associated with diabetic microangiopathy [19]. fMRI takes advantage of the change in the magnetic susceptibility of blood because of the transition of oxygenated (HbO_2_) to deoxygenated (Hb) hemoglobin after releasing oxygen to tissues to meet local metabolic needs. Current techniques, such as the blood-oxygen-level dependent (BOLD) technique—the main representative of fMRI—achieve spatial resolutions of one mm^3^ and temporal resolutions of one sec [20]. BOLD has shown reduced cortical and medullary R2* (transverse relaxation rate) values, a finding indicating enhanced renal parenchyma oxygenation, in patients with type 1 diabetes (T1DM) compared to controls. Furthermore, by also recording reduced cortical functional anisotropy (FA) or structural integrity in the same patients, BOLD demonstrates capability in revealing not only functional but also structural changes of the kidney in patients with DN [21]. MRI data demonstrated a good correlation between biochemical parameters of renal function and inflammation. In another study, BOLD-MRI was used to evaluate kidney oxygenation in patients with type 2 diabetes (T2DM). BOLD-MRI revealed renal hypoxia in DN. More specifically, hypoxia is shown to be more prominent in the medulla compared to the cortex region, highlighting the value of relative indices such as the R2* ratio between the medulla and cortex (MCR) [22]. As revealed by the exemplary studies presented above, current findings about renal oxygenation/hypoxia in DN might be controversial, indicating the need for more focused studies [21]. Another fMRI technique, arterial spin labeling (ASL), which employs the water of arterial blood as an endogenous contrast agent, has also been employed in DN. Finally, Mora-Gutiérrez et al. show that ASL reveals: (i) not only the cortical RBF reduction in patients with diabetes, but also (ii) the DN-mediated changes at different stages of CKD, as defined by GFR [22,23] (Figure 1). 

Diffusion-weighted imaging-MRI (DWI-MRI) has been used to investigate the microstructure of the kidney by monitoring the intrarenal water motion and calculating the apparent diffusion coefficient (ADC), a metric of water diffusion and mobility, which, along with FA (an indicator of water motion directionality), can provide invaluable information about renal microstructure and, indirectly, about blood perfusion/microcirculation [24]. Low ADC indicates high renal fibrosis and organ failure and is, thus, well correlated to the severity of DN as reflected in biochemical parameters such as urinary albumin excretion and GFR [25]. DTI (diffusion tensor imaging), another diffusion (yet more comprehensive than DWI) MRI technique, also uses the ADC and FA to detect the increased renal lipid deposition and microstructural changes in patients with early-stage DN [26,27]. 

The use of computed tomography (CT) and especially contrast-enhanced CT is generally avoided in patients with DN due to the toxic effect of intravenous contrast on renal function.

Even if there are already many established technologies for DN imaging, there is always a need for novel ones. For example, super-resolution ultrasound (SRU) imaging has already been used in clinical and preclinical kidney applications to image the kidney microvasculature with a resolution of 100–150 μm while maintaining the penetration depth at 10–15 cm [28,29]. However, the use of SRU, which necessitates the injection of microbubbles, is still limited by critical disadvantages, such as the relatively long scanning times and the strong dependence of the data quality on the concentration and biodistribution of the microbubbles [29]. 

**Figure 2:**
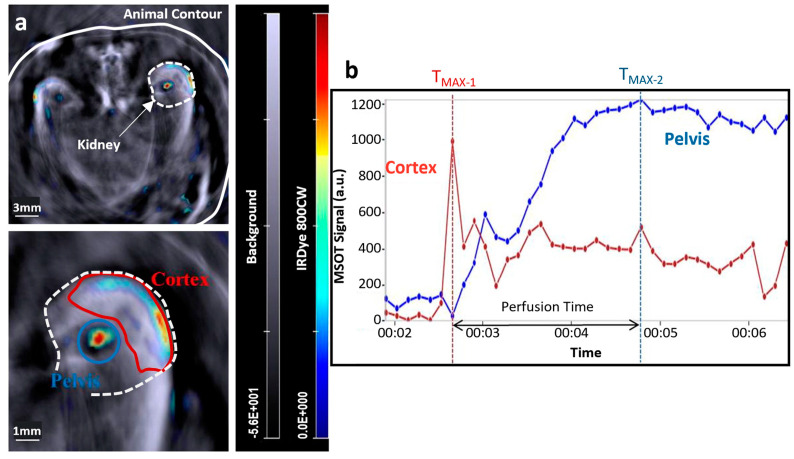
Translational renal imaging with optoacoustics. (**a**) Optoacoustic images of the near-infrared fluorescent IRDye 800CW carboxylate transition from renal cortex to pelvis in a diabetic/obese mouse. The white line region indicates the animal contour, while the white dashed one the kidney. (**b**) Determination of the kidney perfusion time with two kinetic curves showing the fluorescent signal intensities against time. The white dashed line region indicates the pelvis, while the red line indicates the cortex. Adapted with permission from [31].

Another technology that employs fast light pulses and ultrasound is optoacoustic (OAT) or photoacoustic tomography (PAT), with its main representative being MSOT. MSOT is a novel, label-free, non-invasive, high-resolution (~50–250 μm) and real-time technique (25 fps) that allows the measurement of endogenous chromophores, such as HbO_2_, Hb, lipids, and water, and could thus provide information about the renal microstructure and function [30]. The relatively limited penetration depth of MSOT (~2–5 cm, depending on tissue type) would limit its application in examining kidney transplants or relevant preclinical/translational studies. For example, in [31], it is demonstrated that MSOT can provide comprehensive visualizations of intrarenal hemodynamics in a mouse model of diabetes (Figure 2). Of course, MSOT clinical studies on perfusion and oxygenation of soft tissues and organs lying several cm below the skin surface [4,32,33] demonstrate the great potential of MSOT for the visualization of the human kidney in selected groups of patients (e.g., kidney transplant recipients). 

## 3. Diabetic Retinopathy

Diabetic retinopathy (DR) affects the ocular microvasculature and leads to retinal ischemia, as reported by the presence of microaneurysms, local hemorrhages, cotton wool spots, arterial and venous abnormalities, neovascularization in corresponding analyses, and increased permeability of the retina [34]. DR is the most common microvascular complication of diabetes (its prevalence in patients with diabetes is about 27% [35]) and leads to progressive visual impairment and blindness if left untreated [13,36]. Considering its catastrophic consequences and frequently asymptomatic early course, timely DR diagnosis and treatment are an absolute need [13,36]. 

Imaging of the retina via fundus photography (FP) during a dilated eye examination should be conducted at the time of diagnosis for patients with T2DM and within 5 years after the diagnosis for patients with T1DM [13]. FP might have different characteristics even if, in general, a color camera is employed or else optical imaging takes place [37]. For example, traditional systems offer a 30–50° field of view (FOV), while ultra-widefield (UWF) ones provide a 200° FOV [38]. Studies have shown that UWF might improve detection rates compared to traditional approaches [37,39]. In any case, the resolution of retinal details smaller than 20 μm is generally challenging due to normal aberrations of the human eye [40,41]. Fundus cameras are based on the principle of reflex-free indirect ophthalmoscopy to minimize possible reflections [42]. Fundus photography gives direct insights into retinal blood vessels and microanatomic structures, such as the macula, optic nerve, choroid, and vitreous.

Taking into account that the technology of FP is the gold standard method for imaging diabetic retinopathy, there have been many technological developments so far. For example, in [43], the authors employ a white light-emitting diode (LED) confocal device instead of the traditional fundus camera to overcome the frequent red-channel oversaturation. In another study [44], a simple smartphone camera (21.4-megapixel) is shown to be a useful alternative to conventional fundus camera equipment, increasing portability, ease of use, and dissemination. Furthermore, in [45], a red–green–blue (RGB) imaging regime is employed to extract label-free information about the oxygen saturation (SO_2_) and hemoglobin concentration in the fundus, an option that could facilitate early detection of disease. FP has been used not only in static but also in dynamic imaging of the retinal vasculature. For example, Dynamic Vascular Analysis (DVA, Imedos Health GmbH, Jena, Germany) is a technology that employs high-resolution digital FP to assess the endothelial function of the retinal arteries [46]. By employing short light pulses as a trigger (flickering), DVA can provide precise readouts of the light-induced arterial vasodilation to provide insights into the endothelium of the retinal arteries. More specifically, the vasodilatory mechanism induced refers to the activation of the rods and cones, leading to increased metabolism and oxygen demand, which, in turn, is met by the release of vasodilatory nitric oxide (NO) and an increase in local blood flow [47].

**Figure 3:**
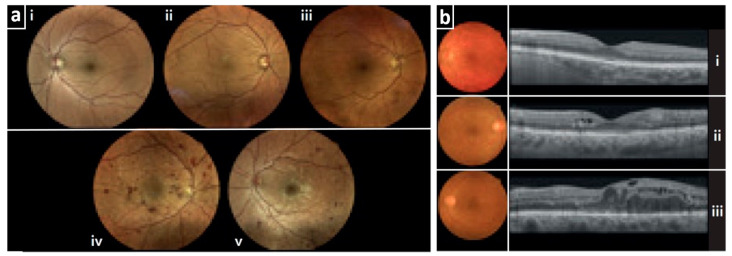
Imaging of retinopathy in diabetes. (**a**) True-color confocal scanning ophthalmoscope images with different stages of diabetic retinopathy (DR): (i) no DR, (ii) mild non-proliferative DR, (iii) moderate non-proliferative DR, (iv) severe non-proliferative DR, and (v) proliferative DR featuring new vessels. (**b**) Color fundus images (round) in the left column (i–iii) and OCT images in the right column (i–iii) showing three stages of diabetic macular edema (DME): (i) no DME, (ii) non-center-involving DME, and (iii) center-involving DME. Adapted with permission from [53].

Apart from the FP, assessment of DR includes a variety of other ocular imaging techniques, such as optical coherence tomography (OCT), OCT-angiography (OCT-A), or ultra-widefield (UWF) imaging, i.e., all innovative non-invasive imaging techniques that can support early diagnosis of DR by identifying relevant disease biomarkers [48]. OCT is a fast and non-invasive imaging method that has become, over the years, a necessary tool for the evaluation of patients with DR [49]. OCT provides high-resolution (5–20 μm) [50,51] cross-sectional images of the retina structure by employing light in the near-infrared range (NIR, 800–1300 μm), and the principle of low-coherence interferometry [52,53] (Figure 3). OCT can identify microanatomic changes, such as the disorganization of retinal inner layers (DRIL), a biomarker that is associated with visual acuity (VA) and can be used to reliably predict future VA in DR [54]. Apart from DRIL, other DR-related biomarkers that can be identified by OCT are hyperreflective retinal and choroidal foci, intraretinal cystoid spaces, bridging retinal processes, increased retinal thickness, and hard exudates [49]. However, even if OCT provides several DR-related biomarkers, it does not give any direct information about retinal ischemia at a microvascular level. 

OCT-A is a technological development of OCT that provides comprehensive visualizations of depth-resolved retinal and choroidal microvasculature without the need for intravenous contrast administration [55]. By providing insights into the retinal deep plexi, OCT-A overcomes a major limitation of fluorescence (fluorescein) angiography (FA), which reaches only the superficial plexus [56]. More specifically, OCT-A can monitor blood flow by comparing consecutive and repeated 2D images (b-scans) at the same cross-section [55]. Thus, OCT-A can directly identify non-perfused capillaries as well as microvascular anomalies, microaneurysms, and neovessels, allowing a better understanding of DR pathophysiology [55]. Several quantitative OCT-A-extracted parameters, including the size of the foveal avascular zone (FAZ), perfusion density, vessel density, vessel length, intercapillary space, vessel diameter index, and fractal dimension [57,58,59], might be associated with the severity and progression of DR [60], demonstrating the potential to be used in the early detection/prediction of DR [61]. 

Fluorescence angiography (FA) enables imaging of blood circulation and, thus, of fundus microvessels. After the intravenous injection of a dye (sodium fluorescein), the circulating dye that reaches the fundus is stimulated with blue light and emits yellow-green fluorescence to be captured by cameras equipped with special filters (an excitation filter of blue light at 465–490 nm and a detection filter of yellow-green light at 520–530 nm [62]). During the examination, several images are taken at different time points, visualizing dynamically different phases of circulation throughout different vascular compartments (e.g., choroidal, arterial, arteriovenous, venous, etc.). DR can visualize retinal neovessels, vascular leakage, and diabetes-related ischemia, such as diabetic macular ischemia (DMI) [60]. Nevertheless, as already mentioned, FA is an invasive technique that requires the injection of a dye, which is associated with rare adverse reactions such as nausea, vomiting, and anaphylaxis [63].

Moreover, the retina offers the opportunity to gain insights into the changes in brain microvascularity, acting essentially as a ‘keyhole’ to several serious conditions, such as neurodegenerative diseases [64], due to strong embryological, morphological, and functional similarities between the two organs [64]. Thus, there are indeed studies showing associations between DR and brain small vessel lesions (detected by MRI), supporting the idea of employing retinal vascular abnormalities as biomarkers to possibly detect and predict brain microangiopathy in diabetes [65].

## 4. Cerebral Microvascular Disease

Many studies have shown that apart from the ‘peripheral’ microvascular complications, including retinopathy, nephropathy, and neuropathy, the microvasculature of the central nervous system is also affected in diabetes [66]. The cerebral microvasculature strongly affects the regulation of critical brain processes. Thus, its deterioration, as observed in DM, predisposes to stroke and cognitive dysfunction. In fact, the risk for ischemic stroke seems to be 2.5 times higher and that for hemorrhagic stroke is 1.5 times higher in patients with DM compared to healthy volunteers [67]. Furthermore, T2DM is associated with a 1.5-times increased risk of developing vascular dementia, while the risk of Alzheimer’s disease in patients with T2DM is increased as well [68]. 

**Figure 4:**
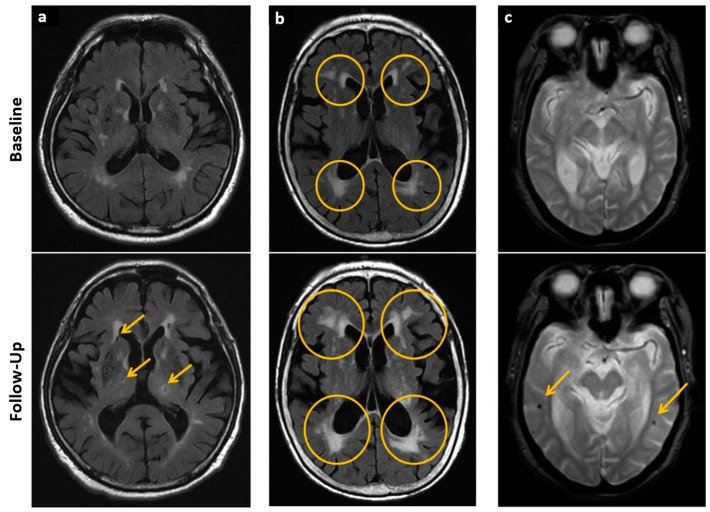
Cerebral imaging. (**a**–**c**) Cerebral small vessel disease implications at baseline (upper row in (**a**–**c**)) and follow-up (lower row in (**a**–**c**)) imaged with MRI. (**a**) Arrows indicate the appearance of new lacunes in the basal ganglia and lateral ventricular anterior horn after 8 years. (**b**) Extending periventricular white matter lesions after 6 years, indicated with circles. (**c**) New microbleeds after 3 years are indicated with arrows. Adapted with permission from [69].

In terms of pathophysiology, hyperglycemia and insulin resistance, hypertension, dyslipidemia/obesity are the main contributors to cerebral microvascular dysfunction in DM [69] (Figure 4). Moreover, not only in patients with DM but also in subjects with prediabetes, the presence of cerebral microvascular dysfunction is prominent, an observation implying that the processes of brain microvascular disease might begin before setting the diagnosis of DM [67].

DM affects the blood–brain barrier and its permeability, altering, thus, the local tissue metabolism and microcirculatory regulation [70]. More specifically, the fronto–temporal cortex [71] and periventricular white matter [70] are the regions frequently damaged in DM. Such changes seem to affect critical cognitive and executive functions [72,73,74]. 

Based on the above, detection and assessment of DM-related brain microvascular disease are of great importance, especially in cases where the underlying condition of the overt clinical phenotype is unclear. DM-related microvascular brain abnormalities can be monitored and characterized using imaging. MRI is the usual technique used for detecting microvascular changes in the human brain. For example, conventional MRI techniques can allow the detection of brain volume reduction due to parenchymal atrophy (cortical and subcortical) in patients with T2DM [75]. Nevertheless, such approaches provide only rough structural information about cerebral changes. 

For this reason, other MRI-based techniques, such as DTI, have been extensively used. DTI has demonstrated high sensitivity to quantify and assess brain microstructural abnormalities in DM. DTI is a quantitative method that reveals alterations in brain microarchitecture, primarily in the white matter region. It detects these changes before macroanatomical abnormalities occur and become prominent with conventional MRI [76,77,78]. DTI measures the motion/diffusion of water molecules within the brain and can be sensitive to intra- and extracellular water orientations [79,80]. Relevant metrics, such as fractional anisotropy (FA), as well as axial (AD), radial (RD), or mean diffusivity (MD), can be calculated and provide a semi-quantitative estimation of water diffusion [77,79,80]. 

Regarding DM, a meta-analysis of 38 relevant studies demonstrated that indeed, DTI can be used as an effective tool to extract biomarkers for the assessment of cerebral microstructural abnormalities in such patients [74]. In particular, the study showed decreased FA and increased AD, RD, and MD within the white and gray matter in DM [74]. 

fMRI, as a method to measure brain tissue functionality by estimating local blood flow, has also been applied in DM. However, in [81], no significant difference in total cerebral blood flow was recorded between patients with T2DM and healthy subjects. 

Moreover, brain microvasculature and function seem to be affected in patients with DM independently of age or type of diabetes. For example, in children with early-onset T1DM, MRI studies showed not only changes in brain volumes but also lower cognitive performance [82]. In addition, dilated perivascular spaces, another MRI-derived index of cerebral microvascular disease, have also been detected in young subjects with T1DM [83,84].

In conclusion, based on the fact that cerebral microangiopathy frequently remains undiagnosed in patients with DM, imaging is crucial for the early detection of this condition and the prevention of its further development. Thus, the evolution of existing techniques and the development of novel ones are considered to be absolutely necessary.

## 5. Skin Microangiopathy and Foot Ulcers

Skin, the largest organ in the human body, forms not only a protective dynamic barrier to exogenous factors, such as mechanical, chemical, and thermal ones, but also a highly sensible sensory organ. Maintenance of skin functional and structural integrity fails in diabetes due to abnormalities of its microvasculature, among other causes. In fact, skin microvascular deterioration occurs early in the natural course of diabetes [85] and frequently before other complications manifest. Thus, features of skin microvasculature may well serve as biomarkers for monitoring diabetes severity and progression. More specifically, both the macro- (e.g., atherosclerosis) and microvascular complications of diabetes may affect the skin microvascular network [66], causing structural and functional changes such as endothelial dysfunction (ED), rarefaction, shrinkage of capillary size and, thus, inadequate tissue perfusion, loss of its health, and formation of chronic wounds with disturbed healing and increased risk for infections: the diabetic foot ulcers (DFUs), which, if remained untreated, could lead to lower limb amputation. More specifically, DFU is a frequent and serious complication of diabetes that is also strongly related to peripheral neuropathy and macrovascular disease, such as peripheral arterial disease [86]. To efficiently identify/assess such changes, optimal non-invasive tools are needed, with imaging and sensing technologies providing several solutions.

Capillaroscopy, a simple optical technique that employs simple digital cameras to acquire high-resolution (approximately 1–6 μm per pixel) photographs of skin capillaries, has been extensively used in diabetes studies. For example, Maldonado G. et al. [87] describe the changes observed in patients suffering from diabetes as the presence of tortuous and dilated capillaries yet with prominent avascular zones within the skin [87]. The advantages of capillaroscopy are its highly disseminated nature and ease of use, as well as its high level of repeatability and reliability [88]. However, capillaroscopy, which is usually used to examine the nailfold region, does not provide tomographic imaging of the whole skin depth.

Laser Doppler flowmetry and imaging (LDF and LDI) are also optical techniques used to monitor skin microvascular perfusion. On the one hand, LDF offers sensing (1D) measurements of the skin’s perfusion by illuminating the skin with laser light. LDF devices are equipped with small sensors that are applied to the skin and can extract information about perfusion over skin volumes of approximately 1 mm^3^ in real time. On the other hand, LDI offers real-time skin perfusion imaging with a spatial resolution of 1 μm and reaching depths of approximately 1–2 mm. However, even if both LDF and LDI are based on the Doppler effect and provide useful information about skin perfusion, the information provided is indirect in the sense that no direct imaging of skin microvasculature at a single-vessel level is provided. LDF and LDI are usually used during functional tests (e.g., post-occlusive hyperemia (PORH), thermal, or iontophoresis tests) where the skin is triggered by means of physical or chemical stimuli and its hyperemic response is recorded and assessed as an index of its functional health. Diabetes, in particular, seems to affect the LDF- and LDI-recorded hyperemic responses of the skin, as shown in relevant studies. For instance, LDF shows a relative reduction in skin microvascular flux over the forearm in response to acetylcholine iontophoresis in patients with T1DM compared to healthy volunteers [89].

LSCI is frequently used in visualizing blood vessels by capturing intravascular blood flow information, as LDI does. More specifically, LDI extracts blood flow information via photodiodes that sense the interference between the light scattered by RBCs (which has a Doppler shift) and that scattered by static tissue light (without a Doppler shift). In contrast, LSCI detects the speckle pattern by means of a CCD camera and relates it to a blood flow model to finally estimate blood flow. LSCI also employs near-infrared light (e.g., 785 nm) and reaches penetration depths of 300 μm. In [90], the authors employed LSCI to image the lower extremity skin in patients with previously diagnosed diabetic foot syndrome, a serious complication of diabetes associated with skin ulcerations and infections due to tissue ischemia and peripheral neuropathy. LSCI revealed clear differences between ischemic and non-ischemic feet, as reflected by diminished microcirculation during functional tests such as PORH and limb elevation tests. Also, LSCI coupled with acetylcholine iontophoresis revealed endothelium in the forearm skin microvasculature in T1DM patients compared to healthy individuals [91]. 

Hyperspectral imaging (HSI) employs light of several different wavelengths within the range of 400–1000 nm [92] to provide multiple images of the same skin region and record, thus, the spectrum of each pixel. The additional spectral information provided can then be used to assign a ‘tissue/molecular label’ for each pixel by comparing the measured spectrum to already known spectra of different tissues/molecules. Thus, biomolecules, such as HbO_2_, Hb, water, and cytochrome c-oxidase (CCO) [93], can be resolved with a resolution of 100 μm. Hyperspectral cameras are usually placed at a distance of 25–30 cm above the skin region to be examined, leaving the skin surface and perfusion undisturbed due to their contactless character [94]. HSI showed reduced perfusion and oxygenation of the skin in patients with diabetes [95]. Furthermore, HSI has shown capability in not only assessing DFU risk but also predicting DFU healing [96].

OCT is capable of not only measuring DR, as discussed in the corresponding section, ‘Diabetic retinopathy’, but also monitoring skin microvasculature and its abnormalities in DM. For example, Argarini et al. visualized and quantified the cutaneous heat-induced changes in the microvasculature of patients with DFU by means of OCT, which directly showed the local changes in microvascular density, diameter, and flow rate [97]. However, OCT was not used to monitor the responses of the different microvascular skin layers (e.g., the papillary and reticular dermis), an option that could possibly provide enhanced pathophysiological insights about diabetes-related microangiopathy. 

**Figure 5:**
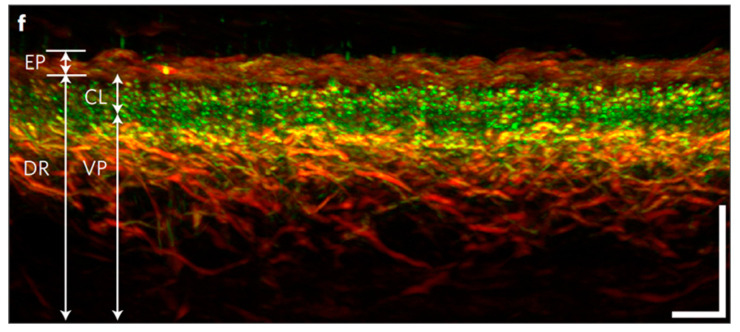
Skin microvascular imaging with RSOM. Tomographic imaging of healthy skin with RSOM, revealing different biological structures and blood vessels according to their spatial frequencies in red (e.g., larger vessels) or green (e.g., smaller vessels). EP: epidermis, DR: dermis, CL: capillary loops and VP: vascular plexus. The scale bars are 500 μm. Adapted with permission from [98].

RSOM, as a novel, non-invasive, label-free optoacoustic imaging technique, can provide highly detailed cross-sectional images of the entire skin depth and all different skin layers (Figure 5) [98,99]. In contrast to MSOT, which is real-time and employs near-infrared light, RSOM needs several seconds (≈45 s) to capture an image and uses green (532 nm) light. With RSOM, the identification of the differences between epidermal and dermal layers is feasible, along with several structures of the dermal papillary region and reticular dermis [100,101]. Recently, He and Fasoula et al. demonstrated that RSOM is capable of providing detailed images of skin microarchitecture and revealed the relationship between RSOM images and diabetes severity by means of several biomarkers. The latter referred to the total number of microvessels and blood volume in the dermal layer, the epidermal thickness, and the optoacoustic signal density [102]. The miniaturized measuring probe of modern RSOM devices and the fact that it acquires numerous signals to produce an image are expected to boost its use as not only an imaging but also optoacoustic sensing technology in the near future. Furthermore, other advantages of RSOM include its relatively low cost as well as its high safety and image quality. 

In general, skin microangiopathy is mainly assessed via the combination of the above-mentioned techniques with functional challenges, such as post-occlusive hyperemia (PORH), thermal triggering, and iontophoresis tests. For example, a meta-analysis based on 13 cross-sectional studies suggests that a dermal microvascular response to local skin heating is significantly reduced in patients with diabetes compared to healthy subjects, as measured by LDF/LSCI [103]. In any case, precise assessment of skin microvascular changes in diabetes is an expanding field that is expected to provide not only the researcher but also the clinician with novel quantifiable tools for improving the management of future patients.

## 6. Diabetic Neuropathy

Diabetic neuropathy (DN) is another common complication with diverse clinical manifestations in patients with DM. DN is mainly classified into two types: diabetic peripheral neuropathy (DPN) and diabetic autonomic neuropathy (DAN). DPN presents with symptoms such as pain, dysesthesia, and numbness, while DAN presents with orthostatic hypotension, tachycardia, sudomotor dysfunction, etc. [13]. Early detection and management of both DN types are critical for minimizing negative sequelae and improving quality of life. DN and its severity are usually monitored by means of physical examinations or questionnaires [104], such as the Douleur Neuropathique 4 questionnaire (DN4) [105], the Brief Pain Inventory (BPI) [106], or the Leeds Assessment of Neuropathic Symptoms and Signs (LANSS) [107], among others. However, imaging and sensing techniques also play an important role in the early detection and objectification of DN. 

Nuclear imaging techniques, such as PET, may detect nerve damage with specific radiotracers, such as C-11-hydroxyephedrine ([^11^C]-HED), which is frequently applied to visualize myocardial sympathetic innervation [108]. Patients diagnosed with diabetic cardiac neuropathy demonstrate significantly decreased radiotracer retention in the apical, inferior, and lateral segments of the heart muscle. The extent of neuronal abnormalities assessed by [^11^C]-HED-PET is associated with the severity of diabetic cardiac neuropathy [109]. Furthermore, PET reveals sympathetic denervation with decreased flow-corrected 6-[^18^F]-fluorodopamine (6-[^18^F]-DA)-derived radioactivity in the lower extremities of patients with painful DN [110], while SPECT seems to be able to quantify the sympathetic innervation in DM by measuring the [^123^I]-metaiodobenzylguanidine ([^123^I]-MIBG)-uptake by the myocardial tissue [108].

One of the results of gastrointestinal neuropathy, which characterizes DAN, is gastroparesis (or diabetic gastroparesis, or DGP). DGP, a condition that manifests with gastrointestinal disturbances (e.g., gastric pain, nausea, vomiting, feeling of stomach fullness, loss of appetite, etc.), affects ~5% of patients with T1DM and ~1% of patients with T2DM and is usually diagnosed by means of gastric emptying scintigraphy (GES). GES measures the gastric emptying of a solid radiolabeled meal achieved at four hours following meal consumption [13,111]. In DGP, gastric emptying is delayed. The condition is characterized by a vague pathophysiology yet seems to be associated with DR, an association possibly indicating the presence of a microangiopathic component [112]. 

Another group of technologies showing the capability of elucidating different aspects of DPN is the MR-based ones [113]. According to [114], MRI can reveal the reduction in spinal cord cross-sectional area that occurs early in DPN and, thus, may well be used as an early marker of the condition. In patients with painful DPN, fMRI shows that enhanced limbic and striatal activations correlate with the duration of neuropathic pain, while blood oxygen level-dependent signals in cerebral structures, such as the anterior cingulate cortex and lentiform nucleus, are associated with thermal stimulations [115]. Also, DTI can quantitatively assess nerve integrity via FA values, showing a significant correlation with nerve conduction velocities and serving as an electrophysiologic indicator of myelin sheath damage [116]. In a relevant study, DTI demonstrated efficient diagnostic accuracy in DPN by measuring metrics such as FA and RD of the tibial and common peroneal nerves [117].

Several studies on diabetes reveal the relationship between microangiopathy and DPN [118,119]. In fact, DN is considered a microangiopathic complication of diabetes [12]. Thereby, promising microvascular imaging technologies, such as RSOM and OCT (Figure 5), could offer additional useful options for evaluating the severity of DN [102]. 

Sudorimetry is a newly developed, non-invasive method based on the principle of reverse iontophoresis to measure the electrochemical skin conductance (ESC) of hands and feet. It provides a quick and quantitative assessment of sudomotor dysfunction, an early detectable abnormality in DN [120,121]. Sudorimetry is not only a reliable screening tool for DN [120], but also a promising tool to screen even asymptomatic DPN in patients with T2DM [122]. 

Finally, despite the several imaging techniques available, the gold standard and most widely used method for the diagnosis of DPN in patients with DM is a sensing method that enables the performance of the so-called nerve conduction studies (NCS) [123]. By using electrodes applied on the skin, several electrical pulses are sent to specific nerves (e.g., median, tibial, sural, etc.), while another electrode records the ‘reaction’ of the muscles supplied by the nerve under examination [124]. This configuration provides insights into the efficacy of the measured peripheral nerve to transmit electrical signals into the innervated muscles. Even if NCS is a quantifiable and sensitive method, it remains time-consuming and requires highly trained personnel in order to be precisely applied [125].

## 7. Cardiac Microvascular Disorders

Cardiac microvascular disease is a serious complication of diabetes [126]. In fact, coronary microcirculation plays a crucial role in regulating coronary blood flow to match tissue and body metabolic demand [127] and might be impaired in patients with diabetes. Over the last decades, the use of several imaging modalities, either non-invasive or invasive, has attempted to shed light on cardiac microvascular disease or coronary microvascular dysfunction (CMVD).

For example, transthoracic echocardiographic Doppler recording (TTE-DR) of coronary blood flow (CBF) may well be employed as the first tool in the clinical routine to detect coronary microvascular dysfunction. With this method, the coronary microvascular dilatator function is calculated as the ratio of diastolic CBF velocity at peak vasodilation to CBF velocity at rest [128]. The method is based on measuring the blood velocity and the increase in coronary artery diameter as a response to an intracoronary injection of an endothelium-dependent vasodilator, such as adenosine [129]. Even if the method is US-based, it is invasive, limited mainly to the assessment of the left anterior descending artery (since the other coronary arteries are difficult to image with US), and not sensitive enough to detect mild coronary microvascular dysfunction [128]. Furthermore, the method provides only indirect information regarding the microvascular function in the heart muscle by assessing the changes in the coronary (macro)vessels and not by providing direct imaging of the cardiac microvasculature. 

Myocardial contrast echocardiography (MCE) can also be used to assess cardiac microcirculation and tissue viability. MCE is US-based and necessitates the intravenous injection of gas-filled microbubbles, which are diffused within the cardiac microvascular bed, destroyed by means of high-energy US, and enable, therefore, the assessment of heart microcirculation via the calculation of two parameters: the mean blood flow velocity and blood volume [130]. MCE has also been used in patients with T2DM, where it demonstrated the capability of detecting myocardial perfusion abnormalities in asymptomatic individuals [131]. Of course, MCE, like any other technique, is characterized by advantages and limitations. Even though it seems to have higher sensitivity than SPECT in detecting microvascular disease in the heart, it employs microbubbles that could potentially occlude the coronaries and can be highly affected by pulmonary motion [132,133]. Another US-based technique called ‘strain rate imaging’ allows the detection of subclinical myocardial dysfunction, which occurs when left ventricular ejection fraction is still preserved. The method has also been applied in patients with diabetes, showing that functional parameters such as myocardial blood flow reserve and resting peak systolic strain rate are lower in T2DM patients compared to healthy subjects, even in the subclinical stages of the disease [134,135]. However, even if strain rate imaging was introduced almost two decades ago, it is still not fully used in everyday practice because image quality might be affected by many different factors and artifacts [136]. 

Moreover, X-ray-based coronary angiography may well be combined with other methods, such as thermodilution, intracoronary Doppler wire flow measurements, and the gas washout method, in order to assess coronary microvascular functions by quantifying CBF and coronary flow reserve (CFR) [126]. Because of its invasive nature, as well as the fact that it requires the injection of possibly toxic contrast agents and the use of ionizing radiation, coronary angiography is not generally proposed for routine use in the assessment of coronary microvascular function.

**Figure 6:**
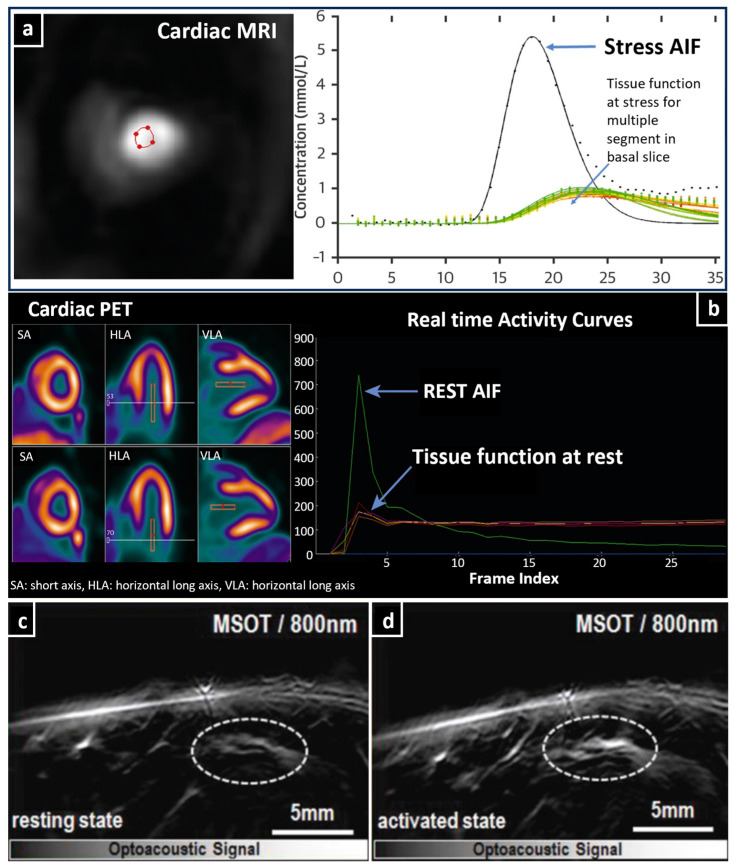
Cardiac and adipose tissue imaging. (**a**) Cardiac magnetic resonance (CMR) imaging to measure stress, Arterial input function (AIF), and tissue function (TF) curves. (**b**) Stress–rest perfusion imaging with PET to obtain AIF and myocardial time-intensity curves. (**c**,**d**) Imaging of supraclavicular region with MSOT in 800 nm, showing BAT activation before and after 20 min of cold exposure. The white dashed line regions indicate a group of blood vessels which are dilated after BAT activation. Adapted with permission from [139,140,143].

In the last few years, other tomographic imaging modalities, such as MRI, PET, and SPECT, have been intensively used in the assessment of cardiac disease. Such techniques employ different tracers and can assess left ventricular function and monitor myocardial blood flow (MBF) in health and disease [137]. Generally, MBF quantification requires the calculation of the arterial input function (AIF), which briefly describes the concentration of the contrast agent (e.g., radiotracer in PET, gadolinium in dynamic-susceptibility contrast (DSC)-MRI, or even blood in the label-free ASL-MRI), which enters and perfuses the myocardium over time [138,139,140] (Figure 6a,b). 

Regarding nuclear imaging techniques, a cross-sectional study showed that coronary flow reserve measured by rubidium-82 (82Rb)-PET-CT is significantly reduced in patients with diabetes, especially in those where albuminuria is also present, a finding possibly indicating that in diabetes a common microvascular impairment might occur in multiple microvascular beds [141]. In another study, technetium-99m (99mTc)-SPECT was used to assess the regional myocardial perfusion reserve in patients with T2DM. The study shows that microvascular dysfunction is uniformly distributed throughout the walls of the left ventricle and usually occurs independently of reversible perfusion defects [142]. 

The variety of the abovementioned studies and techniques showcases the strong role of microvascular components in the pathophysiology of diabetic cardiomyopathy. Thus, even more focused imaging studies are required to further clarify the mechanisms behind this condition beyond the traditional microvascular complications of diabetes, such as the DR, DN, and DPN.

## 8. Microvascular Dysfunction in Skeletal Muscle

The microvasculature of skeletal muscle, a highly dynamic and active tissue, is also affected in patients with diabetes. Individuals with diabetes and insulin resistance show abnormalities in muscle morphology, including changes in muscle fiber composition and low capillary density [144]. In addition, diabetes also affects the microvascular architecture of skeletal muscle in a way that alters the connectivity of the capillary network [145]. In one study, a sample of the quadriceps muscle was taken by needle biopsy for electron microscopic examination, and it was shown that the width of basement membrane capillaries is thicker in patients with diabetes compared to healthy subjects [146]. 

Imaging techniques allow us to better understand the diabetes-related microvascular changes of skeletal muscle in a non-invasive way. For example, contrast-enhanced ultrasound (CEUS) perfusion imaging has been used to evaluate skeletal muscle perfusion (poor in diabetes) in diabetes by measuring microvascular blood volume and capillary responsiveness to insulin [147,148]. Nevertheless, even if CEUS comes with advantages, such as the avoidance of non-ionizing radiation and high portability, it still requires the administration of contrast agents (microbubbles) and advanced image analysis schemes [149]. 

MSOT, which has been recently used in several muscle perfusion and oxygenation studies [150,151], overcomes the limitation of using contrast agents by taking advantage of the strong intramuscular presence of hemoglobin to assess muscle perfusion and oxygenation. Based on its characteristics, MSOT shows great potential to serve as a tool for evaluating the microvascular changes observed within the skeletal muscle in diabetes.

Near-infrared spectroscopy (NIRS), a label-free optical technique utilizing near-infrared light to track Hb and HbO_2_ in tissues, has been widely used in muscle perfusion studies. NIRS probes are light and can be easily applied to the skin surface to measure muscle oxygenation/oxygen kinetics even during exercise. In a relevant study, NIRS showed that patients with T2DM demonstrate early limitations of O_2_ extraction and decreased capacity of HbO_2_ dissociation during cycling exercise compared to healthy volunteers [152]. Of course, NIRS, as a one-dimensional (1D) sensing technique, cannot provide comprehensive high-resolution images of the skeletal muscle, as, for example, MSOT does, while providing information on only a small volume of muscle without visualizing what happens within other muscle regions [153]. 

BOLD-MRI has also been exploited to evaluate peripheral microvascular function in skeletal muscle [154]. Regarding diabetes, a study explored the changes in muscle microvascular function in patients with early (T1DM) and late (T2DM) but short-duration (<10 years) diabetes when measuring the BOLD response following isometric exercise. Results showed that the BOLD-extracted microvascular response was not impaired in such individuals but was negatively correlated with age [155]. 

Moreover, nuclear medicine techniques have also been employed in diabetes-related muscle imaging studies. Specifically, 99mTc-MIBI (Sestamibi) scintigraphy is capable of providing dynamic data on muscle perfusion and monitoring the microcirculation of muscle. In relevant studies, patients with diabetes demonstrate substantially decreased maximum 99mTc-MIBI readouts and peak times of the lower extremity muscle perfusion [156].

Muscles, just like the skin and adipose tissues, offer the great advantage of being easily accessible for imaging and sensing studies. Furthermore, considering the important role of skeletal muscle in the pathophysiology of diabetes, the already large number of relevant imaging and sensing studies is expected to rise in the next few years.

## 9. Microvascular Dysfunction in Adipose Tissues

Adipose tissues are strongly vascularized and metabolically active [157]. In fact, each adipocyte is in close contact with at least one capillary, as shown in microscopy studies [158]. Adipose tissues, mainly divided into white adipose tissue (WAT) and brown adipose tissue (BAT), are largely affected by diabetes. Both tissue types have an impact on glycolipid metabolism and insulin resistance observed in patients with T2DM [159]. Moreover, insulin resistance presented in patients with T2DM is associated with visceral obesity of the central type [160].

Apart from the muscle, CEUS has been applied to examine the dynamics of the microvascular volume in the abdominal subcutaneous WAT after the oral consumption of 75 g glucose. CEUS measurements were combined with WAT blood flow readouts conducted via the ^133^Xenon washout technique. The study shows that both postprandial blood flow and microvascular volume in abdominal subcutaneous WAT are impaired in overweight T2DM patients [161]. Moreover, in another study, CEUS was employed to assess the microvascular function of adipose tissue in T2DM patients, revealing a connection between the degree of obesity and impairment of adipose tissue microvascular function, mainly due to a reduction in capillary density [162].

As already mentioned, MSOT, by employing its unique capability to extract information from hemoglobin, is an imaging modality with great potential to measure the microvasculature of adipose tissues in different scenarios. This unique potential is showcased in a study that employed MSOT in order to detect the hemodynamic changes in the BAT of humans by quantifying markers such as Hb and HbO_2_ over time and following the activation of BAT [7,143] (Figure 6c,d).

Apart from the other organs/tissues mentioned before, MRI has also shown utility in investigating adipose tissue along with cardiac microvasculature in a study including overweight and lean patients with T2DM and overweight and lean healthy volunteers. Epicardial, visceral, and subcutaneous adipose tissues were measured, and MBF was quantitatively estimated. It was revealed that the global stress MBF is reduced in overweight patients with T2DM, who also had significantly higher visceral adipose tissue compared to the other groups [163]. 

This kind of studies where more than one tissue type is examined with a single test and technology, highlights even more the importance of imaging in investigating not only single-tissue changes, but also possible interrelationships among different tissues in the natural course of complex diseases such as diabetes. 

## 10. Diabetic Pulmonary Microangiopathy

Diabetes also affects the pulmonary microvessels, as revealed by histological analyses of the lung parenchyma, where thickening of the basal membrane in the lung capillaries and increased density of the pulmonary microvessels are observed [164,165]. The abovementioned microvascular abnormalities, along with the decreased parenchymal elasticity due to thickening of the alveolar wall, may lead to pulmonary dysfunction, as measured by functional lung tests. 

Spirometry, a characteristic example of such a functional test, measures, essentially, the volume of exhaled air over time. There are multiple spirometer technologies, such as ultrasonic and differential pressure ones [166]. For example, ultrasonic technology is based on the Doppler effect and records the transit time of ultrasound pulses through the inspired and expired air to provide information about the air velocity/flow over time. In any case, spirometry is a simple method that can provide several functional biomarkers, such as the forced vital capacity (FVC), forced expiratory volume in one second (FEV_1_), forced expiratory ratio (FEV_1_/FVC), slow vital capacity (SVC), and maximal mid-expiratory flow (MMEF) [167], to quantify lung function. In [168], patients with diabetes but no pulmonary disease were examined using spirometry. Results showed a clear reduction of FVC, FEV_1,_ and SVC in patients compared to matched healthy volunteers. Furthermore, in [169], reduced lung volumes and airflow limitation were not only detected in patients with T2DM but also correlated with the degree of (poor) glycemic control. Apart from spirometry, lung functional status can also be assessed by means of the diffusing capacity of the lungs for carbon monoxide (DLCO), a marker of the efficiency of the lung to transfer gas from inhaled air to the circulating blood of the pulmonary capillaries. DLCO requires from the patient 10 s of breath-holding, a step that is, however, much better tolerated than the forced exhalation required in spirometry tests [170]. Briefly, the patient inhales air with a small amount of carbon monoxide (CO) and a tracer gas (e.g., methane or helium). After the 10 s breath-holding period, the patient is asked to exhale abruptly and completely the inhaled air mixture. Exhaled gas is directly analyzed in order to calculate how much of the CO and tracer gases were finally absorbed. DLCO is then estimated based on this result, with reduced DLCO (<40–60%) indicating disease. Several studies [171,172,173] have shown reduced DLCO in relevant patients, associating diabetes with pulmonary microangiopathy. But apart from sensing, imaging might also give useful insights into diabetes-related pulmonary microangiopathy. Perfusion chest computed tomography (pCT), in particular, has shown great capability for monitoring changes in CT perfusion parameters throughout the course of diabetes [174]. pCT is capable of visualizing not only the anatomy but also the physiology/function of vessels and tissues by estimating perfusion parameters such as the blood volume (BV), blood flow (BF), mean transit time (MTT), time-to-peak (TTP), and permeability surface (PS) [175]. In fact, diabetes seems to be associated with an increase in the abovementioned perfusion parameters within the lungs, as revealed by chest pCT [174,175]. Moreover, a novel echocardiographic biomarker has also been used in the assessment of pulmonary microvascular disease in diabetes. The biomarker, which is used to quantify the pulmonary transit of agitated contrast bubbles (PTAC), shows that the number of bubbles traversing the pulmonary circulation to reach the left ventricle is reduced in patients with diabetes, especially in those with other coexisting microvascular complications [176]. However, despite its pathophysiological manifestations revealed by targeted examinations, pulmonary microangiopathy does not always lead to clinically overt pulmonary dysfunction.

## 11. Conclusions and Future Steps

Diabetic microangiopathy is a common condition affecting different organs and tissues and cause serious diseases that decrease the quality of life and may lead to death. Early detection and optimal management of diabetic microangiopathy provide an opportunity to take action, possibly delay or prevent progression to further complications. Sensing and imaging of relevant organs and tissues may enable the detection and characterization of such microvascular changes in an easy, convenient, and informative way for the patient.

US and CDI, based on their safety, low cost, and easy operation, are initially applied to the diagnosis of CKD in clinic work. LDF and LSCI are commonly used for the measurement of skin microvasculature by mapping blood flow. These methods are easy and practical for clinical measuring, monitoring, and investigating processes, but with their limited resolution, they cannot visualize and quantify the microvascular anatomy. MRI is an effective imaging technique to measure the abnormalities of the brain. And fMRI sensitively visualizes physiological functions inside the organs and tissues. DTI is another form of MRI with featured parameters that can detect alterations in renal lipid deposition, nerve integrity, and cerebral microstructure. All of these MRI methods are non-invasive, repeatable, and without ionizing radiation; however, they are time-consuming and limited to clinical application in patients without metal implants. PET and SPECT with different specific radiotracers are capable of detecting cardiac microvascular disease and sympathetic denervation and providing high-resolution images. However, PET and SPECT, depending on radiotracers, are expensive, radioactive, and time-consuming in clinic implementation. OCT and its advanced modalities, as noninvasive, and easily applied, entirely safe techniques providing cross-sectional and high-resolution imaging, have been the common and invaluable imaging modality in retinopathy, and OCT also has the feasibility of detecting skin microvascular dysfunction. 

Apparently, it is clear that each technique comes with its own specific strengths and limitations. The fact that numerous techniques have been suggested or introduced in clinical practice so far demonstrates more their complementarity than the adequacy of each one as a stand-alone technique for use in diabetic microangiopathy. Thus, there is still an imperative need for the development of novel sensing and imaging techniques to offer access to condition-specific biomarkers for better diagnostics and stratification of future patients.

## Figures and Tables

**Table 1:** Characteristics of the main techniques employed in the assessment of diabetes-related microangiopathy.

	Imaging Depth	Spatial Resolution	Structural Information	Functional Information	Dynamic Readouts	Ionizing Radiation	Speed of Scan	Equipment Portability	Equipment Cost
US	several cm	medium	yes	yes	yes	no	real-time	medium	low
MRI	whole body	medium	yes	yes	yes	no	several min	low	high
CT	whole body	medium	yes	yes	no	yes	several min	low	high
MSOT	several cm	high	yes	yes	yes	no	real-time	medium	medium
NIRS	several cm	low	no	yes	yes	no	real-time	high	low
FP	superficial	high	yes	yes	yes	no	few min	high	low
OCT	few mm	high	yes	yes	yes	no	few min	medium	medium
FA	superficial	high	yes	no	yes	no	few min	medium	medium
CS	superficial	high	yes	no	no	no	real-time	high	low
LDF	few mm	medium	no	yes	yes	no	real-time	high	low
LDI	few mm	medium	no	yes	yes	no	few min	medium	low
LSCI	few mm	high	yes	yes	no	no	few min	medium	medium
HSI	few mm	medium	yes	yes	no	no	few min	medium	medium
RSOM	few mm	high	yes	yes	no	no	one min	medium	medium
PET	whole body	low	yes	yes	no	yes	several min	low	high
SPECT	whole body	low	yes	yes	no	yes	several min	low	high

US: Ultrasound, MRI: Magnetic Resonance Imaging, CT: Computed Tomography, MSOT: Multi-Spectral Optoacoustic Tomography, FP: Fundus Photography, OCT: Optical Coherence Tomography, FA: Fluorescence Angiography, CS: Capillaroscopy, LDF: Laser-Doppler Flowmetry, LDI: Laser-Doppler Imaging, LSCI: Laser Speckle Contrast Imaging, HSI: Hyperspectral Imaging, RSOM: Raster-Scan Optoacoustic Tomography, PET: Positron Emission Tomography, SPECT: Single Photon Emission Computed Tomography.

## Data Availability

Not applicable.
